# The Selective Acetamidine-Based iNOS Inhibitor CM544 Reduces Glioma Cell Proliferation by Enhancing PARP-1 Cleavage In Vitro

**DOI:** 10.3390/ijms20030495

**Published:** 2019-01-24

**Authors:** Marialucia Gallorini, Cristina Maccallini, Alessandra Ammazzalorso, Pasquale Amoia, Barbara De Filippis, Marialuigia Fantacuzzi, Letizia Giampietro, Amelia Cataldi, Rosa Amoroso

**Affiliations:** Department of Pharmacy, University “G. d’Annunzio”, Chieti-Pescara, via dei Vestini, 31, 66100 Chieti, Italy; marialucia.gallorini@unich.it (M.G.); alessandra.ammazzalorso@unich.it (A.A.); pasquale.amoia@unich.it (P.A.); barbara.defilippis@unich.it (B.D.F.); marialuigia.fantacuzzi@unich.it (M.F.); letizia.giampietro@unich.it (L.G.); amelia.cataldi@unich.it (A.C.); rosa.amoroso@unich.it (R.A.)

**Keywords:** chemoresistance, iNOS, inhibition, Nrf-2, Glioma, PARP-1, MAPK

## Abstract

Gliomas are the most aggressive adult primary brain tumors. Expression of inducible Nitric Oxide Synthase has been reported as a hallmark of chemoresistance in gliomas and several studies have reported that inhibition of inducible Nitric Oxide Synthase could be related to a decreased proliferation of glioma cells. The present work was to analyze the molecular effects of the acetamidine derivative compound 39 (formally CM544, *N*-(3-{[(1-iminioethyl)amino]methyl}benzyl) prolinamide dihydrochloride), a newly synthetized iNOS inhibitor, in a C6 rat glioma cell model. There is evidence of CM544 selective binding to the iNOS, an event that triggers the accumulation of ROS/RNS, the expression of Nrf-2 and the phosphorylation of MAPKs after 3 h of treatment. In the long run, CM544 leads to the dephosphorylation of p38 and to a massive cleavage of PARP-1, confirming the block of C6 rat glioma cell proliferation in the G1/S checkpoint and the occurrence of necrotic cell death.

## 1. Introduction

Malignant gliomas are highly lethal brain tumors with poor prognosis for patients. The current treatment of glioma consists of maximal surgical resection of the tumor, followed by concurrent chemotherapy (Temozolomide, TMZ) and radiation [1]. Chemotherapy resistance is a major cause of treatment failure in patients with malignant brain tumors, and understanding tumor biology and the resistance mechanisms is essential to enhance chemotherapy methods [2].

**Figure 1:**
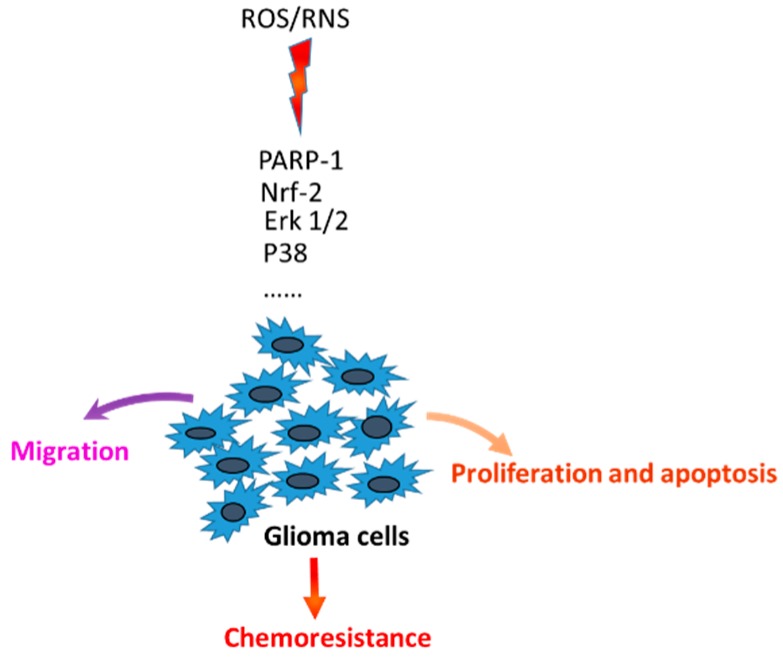
Mechanisms of glioma cells malignancy. ROS and RNS are involved in glioma cells malignancy through the activation of different cellular mediators.

In general, high levels of Reactive Oxygen Species (ROS) and Reactive Nitrogen Species (RNS) are highly involved in the malignancy of gliomas, as well as in chemoresistance due to the activation of different signaling mediators, such as poly(ADP-ribose)polymerase (PARP-1), Mitogen-Activated Protein Kinases (MAPKs), nuclear factor erythroid 2-related factor 2 (Nrf-2) and many others (Figure 1).

Endogenous Nitric Oxide (NO^●^) is a short-lived free radical serving a number of physiological functions [3], synthesized through the enzymatic conversion of L-Arg and molecular oxygen to L-Citr by Nitric Oxide Synthases (NOSs). It has been found that an excess of NO^●^ could cause the generation of RNS, such as the peroxynitrite ion (ONOO^─^) from superoxide anions, which inhibits p53 in malignant gliomas [4]. In particular, studies illustrating the inhibition or genetic deletion of endogenous NOSs support a tumor-promoting role of the inducible isoform of NOS (iNOS) [5,6,7] and numerous studies have suggested a pro-tumorigenic role for NO^●^ in gliomas [8,9]. Studies have reported that iNOS lowered activity is related to a decreased proliferation and invasion of rat glioma cells [10,11].

ROS and RNS, by damaging DNA, can activate PARP-1 which appears a key mediator in malignancy maintenance in various cancers, including Glioblastoma Multiform (GBM) [12]. In general, PARP-1 rapidly uses the substrate β-NAD^+^ to transfer Poly ADP-Ribose (PAR) to itself and to nuclear acceptor proteins. In an effort to resynthesize NAD^+^, the cell consumes its ATP pools and undergoes an energy crisis, resulting in death [13]. Moreover, PARP-1 is a key protein required for cellular functioning and survival, and is cleaved in the caspase-mediated apoptotic cell death [14,15,16].

The induction and maintenance of glioma cell malignancy could also be largely attributed to aberrant MAPK signaling [17]. The MAPKs pathway can be activated by various stimuli, such as growth factors, cytokines and oxidative and nitrosative stress. In particular, gliomagenesis appears to be regulated via crucial signaling mediators such as extracellular signal-regulated kinases (Erks) and p38 mitogen-activated protein kinase (p38). Erk 1/2 activation is implicated in the genesis of a number of human brain tumors [18,19], as well as p38, which is up-regulated in human GBM [20] and is reported to be an apoptotic enhancer upon chemotherapy in various solid tumors.

A further consequence of ROS/RNS high levels is the activation of Nrf-2, which is significantly increased in cytosol and nucleus of glioma cells [21,22]. This could be an important adaptive mechanism that glioma cells develop to regenerate balanced redox homeostasis. Indeed, a major function of Nrf-2 is the activation of downstream target genes coding for a network of enzymatic (e.g., catalase) and non-enzymatic antioxidants (e.g., GSH). [23]. The increased expression of Nrf-2 in glioblastoma was found to protect glioma cells from the killing effects of antitumor therapies, and the blocking of Nrf-2 and related key molecules such as GSH can inhibit glioma cells [24,25,26].

**Figure 2:**
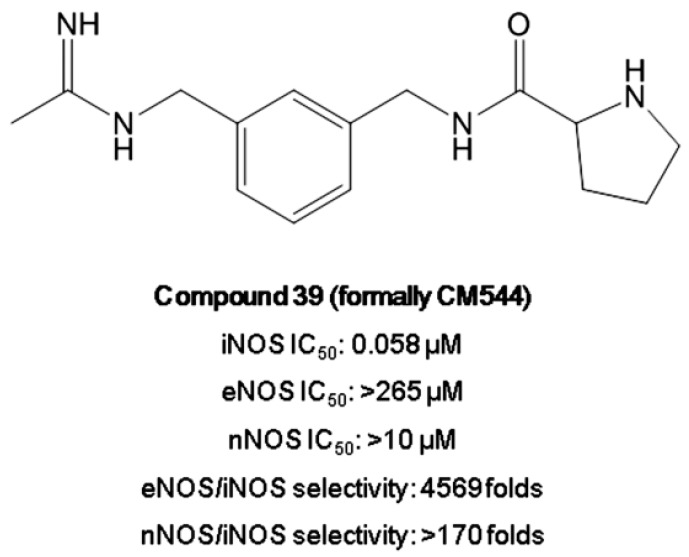
Compound 39 (formally CM544) chemical structure and activity towards NOSs.

As part of an ongoing project on the research of new NOSs modulators [27,28,29,30], we disclosed *N*-(3-{[(1-iminioethyl)amino]methyl}benzyl)prolinamide dihydrochloride (compound 39, formally CM544, Figure 2), a new potent iNOS inhibitor, showing high selectivity with respect to the constitutive endothelial and neuronal NOSs (iNOS IC_50_ = 0.058 μM; eNOS/iNOS selectivity = 4569 folds; nNOS/iNOS selectivity > 170 folds, Figure 1) [31]. From the chemical viewpoint, it is an acetamidine bearing a proline moiety designed with the aim to exploit the subtle differences between the NOSs active sites.

Based on its promising in vitro activity, CM544 was evaluated on rat astrocytes and C6 rat glioma cells, confirming the elevated selectivity towards iNOS, being the compound ineffective on normal astrocytes as they do not express the inducible isoform of the enzyme [31]. Notably, we showed that the exposition of C6 rat glioma cells to 1.5 mM of CM544 arrests the cell cycle at the G1/S phase checkpoint at 1.5 mM, laying the grounds for further molecular studies in vitro.

In the present work, CM544 was investigated in depth to study the molecular response of C6 rat glioma cells to this agent. In particular, ROS and RNS production was analyzed, as well as oxidative and nitrosative stress-related molecular downstream, to clarify the molecular pathway underlying the biological effect of CM544.

## 2. Results

### 2.1. CM544 Influences the Expression of iNOS after Short Times of Exposure and is Ineffective on the Release of Free NO^●^

**Figure 3:**
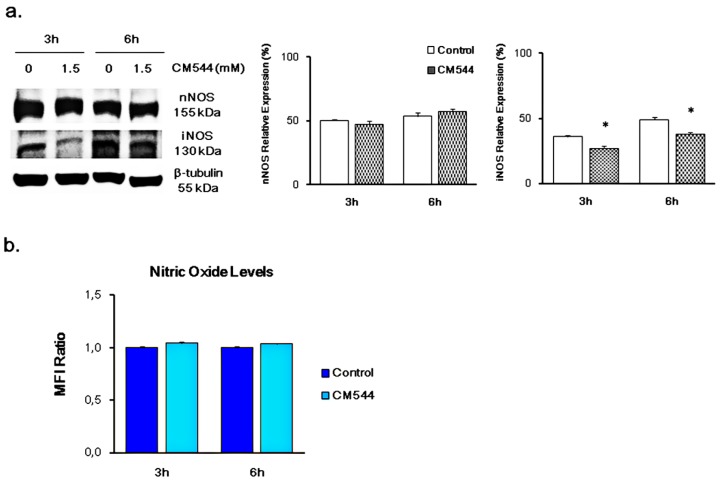
Expression of iNOS and generation of NO in C6 rat glioma cells in the presence of CM544. (**a**) Representative protein bands of iNOS obtained by Western blot analysis. β-tubulin expression was used as protein content marker. Results from one of three independent experiments are shown. Densitometric values are expressed as percentages of the integrated optical intensity of iNOS bands normalized on β-tubulin. * *p* < 0.05 treated vs. Control. (**b**) Bars represent median values (± SD) calculated from individual histograms (*n* = 3). Values are expressed as the MFI Ratio of the control (untreated cells).

The effect of CM544 on the expression of NOSs was evaluated by means of immunoblotting after 3 and 6 h of exposure to 1.5 mM. The expression of the constitutive isoforms nNOS was not affected by the treatment, being the protein bends similar over the time of the culture and the treatments (Figure 3a). In contrast, iNOS was clearly down-regulated after 3 h of exposure to CM544 with respect to untreated cells (27% vs. 36%). After 6 h, iNOS expression increased in untreated cultures, having slightly decreased after exposing cells to CM544 with respect to a 3 h treatment (Figure 3a).

The amount of free intracellular NO^●^, quantified to analyze the influence of CM544 on the release of this radical by C6 rat glioma cells after 3 and 6 h, was not significantly influenced by the treatment (Figure 3b).

### 2.2. CM544 Enhances the Generation of Reactive Oxygen/Nitrogen Species and Induces the Expression of Nrf-2

For the detection of reactive oxygen/nitrogen species generated by glioma cells exposed to CM544, we used a flow cytometrical detection by means of the chemical reporter CM-H2DCFDA. CM-H2DCFDA is a non-fluorescent dye that passively diffuses into cells, where its acetate group is hydrolyzed by esterases to the corresponding acid and the chloromethyl group reacts with glutathione and other thiols. Subsequent oxidation yields the fluorescent adduct 2′,7′-dichlorofluorescein (DCF). Increased intensity in fluorescent DCF could reflect the detection of certain reactive oxygen and nitrogen species, including nitroxidative stress [32].

**Figure 4:**
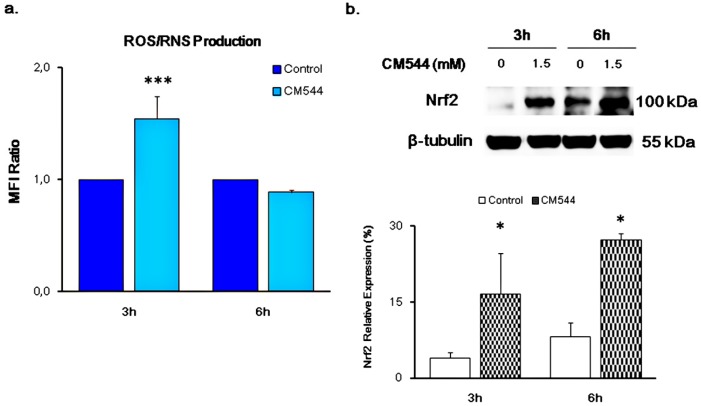
Generation of Reactive Oxygen/Nitrogen Species (ROS/RNS) and expression of Nrf-2 in C6 rat glioma cells in the presence of CM544. (**a**) Bars represent median values (± SD) calculated from individual histograms (*n* = 3). Values are expressed as the MFI Ratio of the control (untreated cells). *** *p* < 0.001 treated vs. Control. (**b**) Representative protein bands of Nrf-2 obtained by Western blot analysis. β-tubulin expression is used as protein content marker. Results from one of three independent experiments are shown. Densitometric values are expressed as percentages of the integrated optical intensity of Nrf-2 bands normalized on β-tubulin. Nrf-2: nuclear factor (erythroid-derived 2)-like 2. * *p* < 0.05 treated vs. control (untreated cells).

As shown in Figure 4a, increased intracellular levels of oxidative and nitrosative stress were widely and consistently observed in glioma cells exposed to 1.5 mM of CM544 for 3 h. However, CM544 was ineffective after longer exposure time, being the Mean Fluorescence Intensity (MFI) ratio of a 6 h treatment comparable to the one of UC. Early exposures (3 h) of CM544 also triggerred Nrf-2 expression and the increment was further enhanced after 6 h (16.7% and 27.3%, respectively) (Figure 4b).

### 2.3. Modulation of MAPKs and p53 in the Presence of CM544

**Figure 5:**
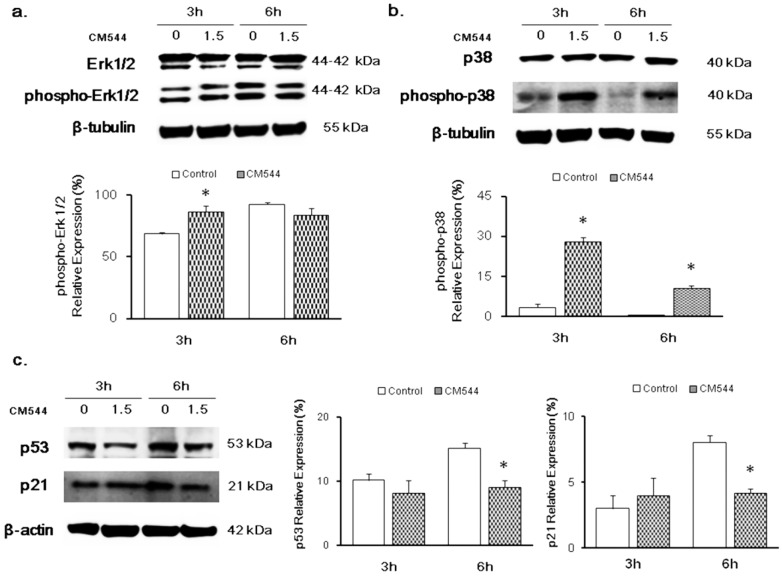
Modulation of MAPKs and p53-p21 in C6 rat glioma cells in the presence of CM544. Representative protein bands obtained by Western blot analysis. (**a**) Erk 1/2 and pErk 1/2 protein expression. (**b**) p38 and pp38 protein expression. (**c**) p53 and p21 protein expression. β-tubulin and β-actin expression are used as protein content markers. Typical results from one of three independent experiments are shown. Densitometric values are expressed as percentages of the integrated optical intensity of protein bands normalized on β-tubulin and β-actin. * *p* < 0.05 treated vs. control (untreated cells). ** *p* < 0.01 treated vs. control (untreated cells).

As the MAPK cascade activation is involved in glioma cell proliferation and invasion, the expression of phosphorylated Erk 1/2 and p38 was quantified by immunoblotting. Phospho-Erk 1/2 relative expression slightly increased in the presence of CM544 after short exposure times (3 h) while the ratio between the phosphorylated protein and its full length did not significantly change after a 6 h treatment (Figure 5a). Notably, 1.5 mM of CM544 dramatically influenced p38 activation after 3 h of exposure, being phospho-p38 up-regulated with respect to untreated glioma cells (28% vs. 3.4%). On the contrary, the expression of the activated p38 was halved after 6 h of exposure to CM544, although remaining significantly higher with respect to untreated cultures (10.7% vs. 0.3%) (Figure 5b).

To determine whether the increased oxidative and nitrosative stress induced by CM544 could provoke the modulation of p53 through phospho-p38 regulation, the expression of p53 and its related protein p21 was quantified. p53 was clearly expressed in untreated glioma cells after 3 h of culturing while it was down-regulated in the presence of 1.5 mM CM544. The same effect but to a major extent could be detected after 6 h (Figure 5c). In parallel, the expression of p21 decreased after exposing cells to CM544 for 6 h (Figure 5c).

### 2.4. CM544 Causes PARP-1 Activation after 3 h of Treatment

**Figure 6:**
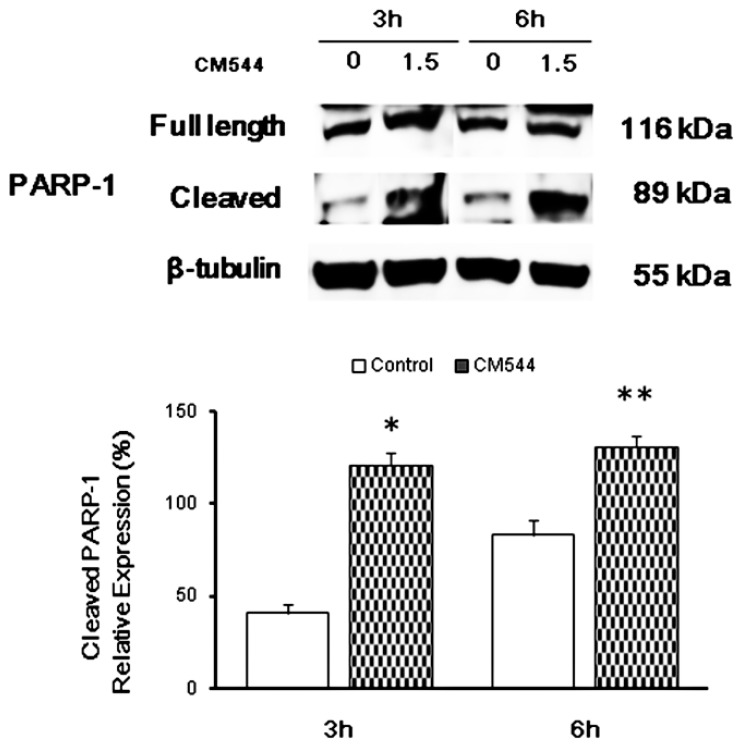
PARP-1 activation in C6 rat glioma cells in the presence of CM544. Representative protein bands of PARP-1 obtained by Western blot analysis. β-tubulin expression is used as protein content marker. Typical results from one of three independent experiments are shown. Densitometric values are expressed as percentages of the integrated optical intensity of protein bands normalized on β-tubulin. ** *p* < 0.01 treated vs. control (untreated cells).

To evaluate the modulation of PARP-1 after oxidative and nitrosative stress occurrence induced by compound 39, the full length and the cleaved counterpart relative protein expression was quantified after 3 and 6 h of treatment (Figure 6). PARP-1 (full length) was well expressed in all experimental conditions, confirming its well-known overexpression in glioblastoma and its involvement in chemoresistance. As regards to cleaved PARP-1, its relative expression was significantly higher after a 3 h treatment with respect to untreated cultures (121% vs. 41%). After a 6 h treatment with CM544 1.5 mM, PARP-1 cleavage increased further as compared to untreated glioma cells (131% vs. 83.3%).

### 2.5. Cell Metabolic Activity and Cell Cycle Progression in Cultures Exposed to TMZ and CM544

**Figure 7:**
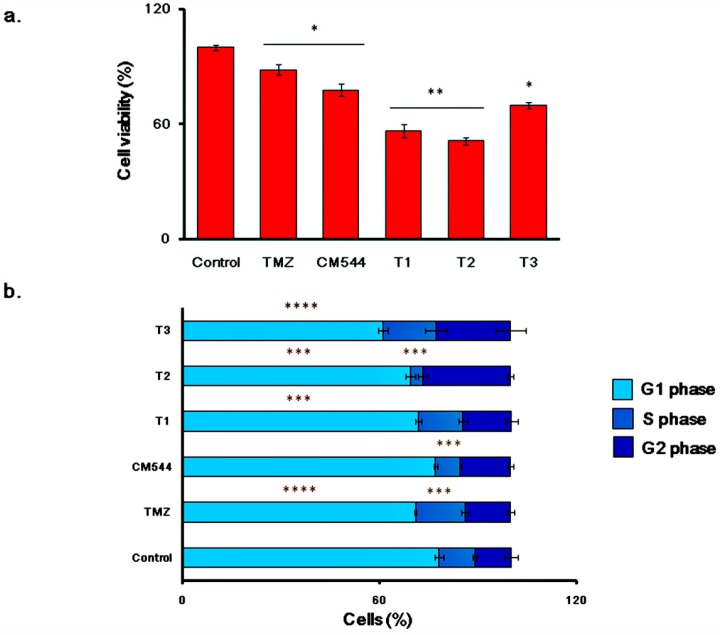
Cell metabolic activity and cell cycle progression of C6 rat glioma cells in the presence of TMZ and CM544. Metabolic activity of C6 rat glioma cells is expressed as the ratio (%) of untreated cells (Control) measured by the MTT test (**a**). Results represent the means ± S.D. (*n* = 9). Control (untreated cells); TMZ (500 µM TMZ 24 h); CM544 (1.5 mM CM544 24 h); T1 (500 µM TMZ 48 h); T2 (1.5 mM CM544 48 h); T3 (1.5 mM CM544 24 h as pre-incubation; 500 µM TMZ + 1.5 mM CM544 24 h). * *p* < 0.05 TMZ, CM544 and T3 vs. Control; ** *p* < 0.005 T1 and T2 vs. Control; ** *p* < 005 T1 vs. TMZ and T2 vs. CM544. (**b**) Cell percentages in the various phases of the cell cycle (G1, S and G2) of C6 rat glioma cells. Results represent the means ± S.D. (*n* = 3). Samples were named as for the MTT assay. *** *p* < 0.002 G1 phase of T1 and T2 vs. Control; *** *p* < 0.002 S phase of TMZ, CM544 and T2 vs. Control; *** *p* < 0.002 S phase CM544 vs. TMZ; *** *p* < 0.002 S phase of T2 vs. T1 and T3. **** *p* < 0.0005 G1 phase of TMZ and T3 vs. Control; **** *p* < 0.0005 G1 phase of T3 vs. TMZ, CM544, T1 and T2.

To compare the biological effects of the commercial chemotherapeutic agent TMZ to CM544, metabolic activity and cell cycle progression of C6, rat glioma cells were analyzed after treating cells with the two agents separately and together (Figure 7). TMZ 500 µM significantly reduced cell metabolic activity after 24 h and 48 h of treatment (88.44% and 56.58% of metabolizing cells for TMZ and T1, respectively; Figure 7a) Notably, 1.5 mM CM544 administered at the same experimental times enhanced cell viability fall, being the percentages of active metabolizing cells assessed at 77.85% and 51.34%, respectively (CM544 and T2; Figure 7a). When cell cultures were pre-incubated with 1.5 mM CM544 for 24 h and then co-treated with CM544 and TMZ for further 24 h, cell metabolic activity was assessed at 69.76% (T3; Figure 7a). As expected, 500 µM TMZ reduced percentage of cells found in the G1 phase with respect to untreated control (71% vs. 78.1%) after 24 h and 48 h (71.93%) (Figure 7b). The administration of 1.5 mM CM544 was slightly effective on the G1 phase after 24 h, exerting its antiproliferative effect at 48 h, as shown by the percentage of cells in the G1 phase assessed at 69.52%. More relevant, CM544 significantly decreased the percentage of cells found in the S phase of the cell cycle with respect to the untreated control after 24 h (7.52% vs. 10.96%) and even more after 48 h of treatment (3.59%). When cells were pre-incubated with 1.5 mM CM544 and afterwards exposed to TMZ and CM544 in parallel (T3; Figure 7b), the percentage of cells in the G1 phase was the lowest value registered, being assessed at 61.14%.

### 2.6. Cell Viability of CM544-Exposed Cells in the Presence of BSO

**Figure 8:**
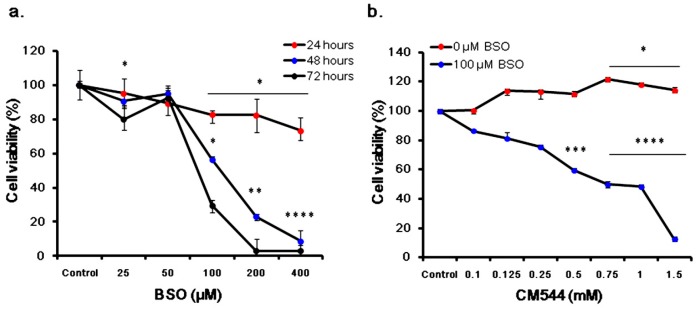
Cell metabolic activity of C6 rat glioma cells in the presence of BSO and CM544. Metabolic activity of C6 rat glioma cells with BSO alone (**a**) and BSO in the presence of CM544 (**b**) is expressed as the ratio (%) of untreated cells (Control = 0 mM BSO or CM544) measured by the MTT test. Results represent the means ± SD (*n* = 9). * *p* < 0.05, ** *p* < 0.01, *** *p* < 0.005 and **** *p* < 0.001 cells treated vs. control (untreated cells).

To confirm the role of the non-enzymatic antioxidant glutathione (GSH) in glioma chemoresistence and in an effort to lower the effective dose of CM544 on C6 rat glioma cells, cell viability was measured through the metabolic activity assay (MTT). The first step of experiments (Figure 8a) was carried out to identify the subtoxic dose useful for further investigations. As shown in Figure 8a, there was a slight but significant dose-dependent decrease of cell metabolic activity after 24 h of treatment. After 48 h, a dramatic fall was registered in the range of 100–400 µM BSO (56.40%, 23.00% and 8.65%, respectively), becoming even more enhanced after 72 h in the same range of concentrations (29.56%, 3.04%, 2.94%, respectively). Since concentrations higher than 100 µM BSO revealed an overmuch cytotoxicity, this concentration was used for the further step of experiments. Figure 8b shows metabolic activity of C6 rat glioma cells treated with loading concentrations (0–1.5 mM) of CM544 alone and in the presence of 100 µM BSO after 24 h. As already shown in our previous work [31], after a 24 h administration of CM544 to C6 rat glioma cells, the percentage of cell metabolic activity increased, being significantly (*p* < 0.01) augmented in the presence of 1 mM and 1.5 mM. By contrast, adding 100 µM BSO to the cultures in the presence of loading concentrations of CM544, dramatically decreased cell metabolic activity after the same exposition time (24 h), being the percentage of metabolizing cells assessed at 59.53% after the administration of 0.5 mM CM544 and 100 µM BSO and at 49.90% and 48.19% with CM544 0.75 mM and 1 mM, respectively.

## 3. Discussion

GBM shows the highest incidence among primary brain tumors exerting a very poor prognosis. Even though clinical treatment standards have reached optimal conditions, the median survival of patients with GBM is approximately 15 months with a five-year survival rate of <4% from the time of diagnosis. Hence, the discovery of additional pharmacological approaches able to ameliorate TMZ effects and radiotherapy is urgently needed [2]. Several studies suggest that NO^●^ and NOSs play a pivotal role in the pathogenesis of GMB and participate in cell response towards chemotherapeutic agents [31]. iNOS expression was found considerably high in glioma cells both promoting and inhibiting actions which have been recently described [3,10].

Our group has recently reported that compound 39 (formally CM544, Figure 2) is a potent and selective iNOS inhibitor, able to reduce C6 rat glioma cell proliferation after preliminary tests in vitro [31]. To confirm the selectivity of this newly synthetized molecule towards its enzymatic target, CM544 was administered to both C6 rat glioma cells and rat astrocytes, where the iNOS is not overexpressed. Our data on metabolic activity show that CM544 exerts no effects on astrocytes over the time of the treatment, whereas it was found to be significantly effective after 48 h and even more after 72 h on C6 rat glioma cells in the range 1–2 mM. Moreover, cytotoxicity data confirm that the decrease in the proliferation rate is necrosis-related, being the LDH release augmented in a time-dependent manner when 1.5 mM CM544 is administered after 24, 48 and 72 h. Based on these results, 1.5 mM of CM544 has been selected for further investigations in vitro. CM544 is an acetamidine bearing an aromatic group separated from a proline moiety by an amidic bond (Figure 3). As reported, these molecular portions are responsible for the binding to the iNOS active site, exploiting the subtle differences among the three NOS isoforms. In particular, the acetamidino group establishes essential bidentate H-bonds. However, this group is responsible for the high polarity of CM544, which shows a clogP of −0.89. It is plausible to assume that the elevated dose needed here to achieve a biological and a molecular effect in the C6 rat glioma cell line is strongly related to the polarity of the molecule.

iNOS activity can be indirectly evaluated by simultaneously measuring its protein expression and the intracellular release of free nitric oxide. Assuming that glioma cells derive from malignant transformed astrocytes [33], it is plausible that the neural isoform of NOS (nNOS) is physiologically expressed in untreated C6 rat glioma cells over the time of the culture (Figure 3a). Clearly, the expression of nNOS was not affected by CM544, whereas iNOS was found decreased after a 3 h treatment. These data suggest that CM544 is bound to the enzyme and prevents its catalytic activity, being the antibody not capable of detecting the iNOS protein.

Our data show that CM544 seems to be ineffective on the release of free NO^●^ after 3 h and 6 h treatment (Figure 3b). Physiologically, NO^●^ reacts faster with O_2_^−^ to produce the highly reactive NOO^−^ rather than with superoxide dismutase (SOD) to result in H_2_O_2_ [5,34]. In this study, we evaluated the production of ROS/RNS by means of CM-H2DCFDA. It is widely known that the CM-H2DCFDA is not a specific probe for a particular oxidant and can be used to monitor both ROS and RNS. Increased intensity of fluorescent DCF could reflect the detection of nitroxidative stress [35,36]. Consequently, CM-H2DCFDA raised fluorescence caused by CM544 after 3 h could be due to the increased levels of both ROS and RNS. The availability of a large amount of O_2_^−^ anions, which are spontaneously transformed into NOO^−^, could be responsible for this effect. In parallel, the increase of Nrf-2 expression is as fast as the induction of ROS/RNS (Figure 4b), demonstrating a direct correlation between oxidative/nitrosative stress occurrence and Nrf-2 activation in CM544-exposed cells. At the same time, Nrf-2 activation can explain the drop of ROS/RNS levels observed after 6 h treatment (Figure 4a). Indeed, the expression of Nrf2 triggers the expression of Glutamate-cysteine ligase modifier subunit (GCLM) and Glutamate-cysteine ligase catalytic subunit (GCLC), which are antioxidant enzymes involved in GSH synthesis and glutathione redutase, glutathione peroxidase and glutathione S-transferase, which are key regulators of GSH utilization. It has been reported that xenobiotics decrease Nrf-2 degradation, followed by nuclear translocation and activation of Antioxidant Response Elements (ARE)-dependent gene expression. In normal cellular conditions, Nrf-2 has anti-cancerous effects due to the induction of ARE-dependent cytoprotective genes expression that protect the cells from oxidative and electrophilic stress [14]. However, in a number of pathological conditions such as GMB, a prolonged activation of Nrf-2, contributes to the development of drug resistance with different molecular mechanisms [36]. Consequently, endogenous ROS levels can be decreased in cancer cells, and this makes the tumors resistant to chemo- and radiotherapy [37,38]. Moreover, it was reported that activation of Nrf-2 induced MGMT (O^6^-methylguanine-DNA methyltransferase), an important player of TMZ-resistance [39]. The increased expression of Nrf-2 after 3 and 6 h CM544 administration confirms a role of this antioxidant regulator in cancer cells oxidative stress-related chemoresistance [40]. On the other hand, iNOS upregulation is also involved in TMZ-resistance [41], and therefore the selective inhibition of this enzyme could be a strategy to improve TMZ efficacy. As a preliminary evaluation of this hypothesis, we compared the effects of TMZ and CM544 on C6 rat glioma cells. Results clearly show that CM544 can reduce cell viability even more than TMZ (77.85% vs. 88.44%, respectively, after 24 h of drug treatment, and 51.34% vs. 56.58% after 48 h; Figure 7a). Interestingly, the co-administration of CM544 and TMZ (T3 in Figure 7) leads to a further reduction of cell viability with respect to TMZ treatment alone (69.76% vs. 88.44%, respectively; Figure 7a). Consistently, a reduction of cells found in the G1 phase of cell cycle progression can be observed after the CM544-TMZ co-treatment (T3, Figure 7b) in comparison with both control and TMZ treated cells, supporting the idea that CM544 could improve TMZ effects. Nevertheless, more experiments in appropriate chemoresistant cell lines are necessary to assess the ability of this new iNOS inhibitor to improve the activity of TMZ.

Two main protein families are known to regulate ROS/RNS-activated signal transduction pathways, namely the MAPKs and the redox sensitive kinases. After being activated via extracellular stimuli, MAPKs phosphorylate their substrates at Ser and/or Thr residues. After activation, phosphorylations by MAPKs lead to the activation of diverse signaling pathways, including proliferation, differentiation, and cell cycle arrest [38]. One of the most crucial pathways involved in gliomagenesis is the MAPK pathway which appears to regulate the genesis and the progression of glioma via crucial signaling mediators such as RAF (Rapidly Accelerated Fibrosarcoma), RAS (Rat Sarcoma), Erk, JNK (c-Jun N-terminal kinase) and p38 [16]. Erk1/2 are members of the MAPK family and they are also important members of an intracellular signaling cascade involved in the development of neurons and glia [38]. It has previously reported that the activation of Erk1/2 by oxidative stress enhance cell viability and proliferation in different types of tumor [39]. The mechanism underlying this activation is still not clear but it has been shown that oxidative aberrations of MAPK signaling proteins could be involved [42]. CM544 significantly increased the expression of phosphorylated Erk1/2 after a 3 h treatment. The augmented activation of Erk1/2 and Nrf-2 could be related to the increased oxidative and nitrosative stress, in an effort for the cells to counteract the effect of chemotherapy. This match with metabolic activity data after 24 h of treatment, when the highest percentage of C6 rat glioma cell proliferation is registered in the range 0.75–1.5 mM [31]. Likewise, phosphorylated p38 was found to have significantly increased after a 3 h treatment with CM544 (Figure 6). However, after 6 h, the amount of activated p38 dramatically decreased. Considering that p38 is a key molecule in pathways underlying malignant transformation and tumor maintenance in gliomas [16], the reduced expression of p-p38 is a first step in describing CM544 cytotoxic effect towards C6 rat glioma cells (Figure 7).

DNA and protein damage induced by ROS/RNS and several chemotherapeutic agents promotes both p53 and PARP activation. Activation of the oncogene p53 is widely known to regulate apoptotic cell death, whereas the cleavage of PARP-1 is related to necrotic cell death occurrence associated with energetic collapse [43]. When p53 is activated, apoptosiscauses activation of caspase, apoptosome and programmed cell death. Depending on the impact of oxidative/nitrosative stress occurrence, these two proteins can either repair the damage or activate cell death pathways [44]. Moreover, the activation of p53 could lead to cell cycle arrest through the transcriptional downregulation of different cell cycle genes. Recently, it was reported that the p53-dependent repression is controlled by the p53-p21-DREAM-E2F/CHR pathway (p53-DREAM pathway) [45]. In our system, C6 rat glioma cells respond to CM554 both down-regulating p53 and p21 after 3 h and 6 h treatment (Figure 5). In parallel, PARP-1 was found activated after 3 and 6 h (Figure 6) confirming our data on cell cycle, which has been found significantly blocked in the S-phase [31].

The understanding of the interaction between key molecules involved in stress adaptive mechanisms and CM544 lays the grounds for further investigations to innovate strategies against CM544-related chemoresistance in an effort to lower the elevated dose administered here. The intracellular pool of GSH is highly abundant and is crucial for the redox cellular homeostasis. Moreover, as a highly reactive thiol, GSH can inactivate xenobiotics, and has been demonstrated to act as a key molecule in glioma chemoresistance [25,46]. In many cell lines, GSH intracellular levels can be depleted by up to 90% through the administration of BSO (Buthionine sulfoximine), an irreversible selective inhibitor of glutamate cysteine ligase (GCL) [47]. We previously reported that the rise of the cell metabolic activity after 24 h of administration of CM544 on C6 rat glioma cells may be due to mechanisms of chemoresistance active in malignant gliomas, such as an increased GSH intracellular pool [31]. In our rat glioma cell model, we demonstrated that exposing cultures to 100 µM BSO in parallel with loading concentrations of CM544 can be a useful tool to improve the experimental set up using lower doses of our newly synthetized compound, for instance in the range of 0.5–0.75 mM.

## 4. Materials and Methods

### 4.1. Materials

All chemicals were purchased from commercial sources and used without further purification. Flash chromatography was performed on silica gel 60 (Merck, Darmstadt, Germany) and TLC on silica gel 60, F254 (Merck, Darmstadt, Germany). Infrared spectra were recorded on a FT-IR 1600 Perkin-Elmer spectrometer (Wellesley, MA, USA). NMR spectra were run at 300 MHz on a Varian instrument (Palo Alto, CA, USA); the solvent was DMSO-d6; chemical shifts (δ) are reported in ppm. Mass spectra were obtained on a Thermofinnigan LCQ Advantage spectrometer (ESI) (San Jose, CA, USA). Elemental analyses were carried out by a Eurovec-tor Euro EA 3000 model analyzer (Pavia, Italy). Compounds had a purity of ≥95%.

### 4.2. Synthesis

The iNOS inhibitor compound 39 (CM544, Figure 2) was synthesized as previously described [31].

### 4.3. Cell Culture

C6 rat glioma cells (ECACC^®^ 92090409, Sigma Aldrich, Milan, Italy) were cultured in HAM’s F-12 supplemented with 1% L-Glutammine, 10% FBS and 1% penicillin/streptomycin (EuroClone S.p.a., Milan, Italy). Cells were maintained at 37 °C and 5% of CO_2_ and sub-cultured as recommended by ECACC^®^.

### 4.4. Generation of NO^●^

Nitrixyte™ probe (Cell Meter™ Fluorimetric Intracellular Nitric Oxide Assay Kit, AAT Bioquest, CA, USA) was used for detecting the production of free NO^●^ in C6 rat glioma cells by flow cytometry. Nitrixyte™ can react with NO^●^ to generate a bright orange fluorescent product that has spectral properties similar to Cy3^®^ and TRITC, as already reported [48,49,50]. Cells were seeded (2.4 × 10^5^/well) in a 6-well tissue culture-treated plate (Falcon^®^, Corning Incorporated, NY, USA) and let to adhere for 24 h. After that, the growth medium was removed and replaced with fresh HAM’s F-12 containing CM544 (1.5 mM final concentration) and 1 µL/mL of 500× Nitrixyte™ Orange and incubated at 37 °C and 5% of CO_2_ in the dark. After 3 and 6 h of exposure, cells were harvested and processed according to the manufacturer’s instructions. The analysis was performed by using a CytoFLEX Flow Cytometer (Beckman Coulter, FL, USA) with a FL2 (PE) detector in linear mode. The experiment was performed in triplicate. Results are expressed as the mean value ± SD of phychoerythrin peak of emission medians obtained analyzing samples with CytExpert software (Beckman Coulter, FL, USA) and are provided to quantify the fluorescent changes in the FL2 channel.

### 4.5. Formation of ROS/RNS

The intracellular production of ROS/RNS in C6 rat glioma cells was detected using 2.5 µM of the oxidation-sensitive probe CM-H2DCFDA (5-(and-6)–chloromethyl-2′,7′-dichlorodihydrofluorescein diacetate) (Life Technologies, Milano, Italy). Cells were seeded in a 6-well tissue culture-treated plate (Falcon^®^, Corning Incorporated, NY, USA) at 2.4 × 10^5^/well and let to adhere for 24 h. Then the C6 rat glioma cells were stimulated with 1.5 mM CM544 for 3 and 6 h. When ROS/RNS are produced, the CM-H2DCFDA is oxidized and an increase in green fluorescence can be detected by a CytoFLEX cytometer with an FL1 detector in linear mode using the CytExpert software (Beckmann Coulter, FL, USA). Each experiment was performed in triplicate. The MFI ratio was obtained by histogram statistics and provided to quantify the ROS production. Results are expressed as mean values ± SD.

### 4.6. Cell Lysis and Protein Extraction

C6 rat glioma cells (2.5 × 10^4^/cm^2^) were first cultured in T25 culture flasks at 37 °C and 5% of CO_2_ for 24 h. Then, cells were incubated with 1.5 mM CM544 for 3 and 6 h. After that, floating and adherent cells were harvested by trypsinization and collected in cold PBS by centrifugation. Cell pellets were washed twice with cold PBS. Lysis buffer (0.5 mL) plus protein inhibitors cocktail (PBS, 1% IgePal CA-630, 0.5% sodium deoxycholate, 0.1% SDS, 10 mg/mL PMSF, 1 mg/mL aprotinin, 100 mM sodium orthovanadate and 50 µg/mL leupeptin) was added and set on ice for 30 min. The lysed pellets were then re-suspended and kept on ice for a further 30 min. Following centrifugation for 15 min at 20,000 *g*, supernatants were collected as whole cell fractions. Protein concentration was determined using a bicinchoninic acid assay (QuantiPro™ BCA Assay kit for 0.5–30 μg/mL protein, Sigma-Aldrich, Milan, Italy) following the manufacturer’s instructions.

### 4.7. Immunoblotting

The C6 rat glioma cell lysates (15 μg/sample) were electrophoresed on a 4–20% SDS-PAGE Gel (ExpressPlus™ 10 × 8, GenScript Biotech Corporation, China) and transferred to nitrocellulose membranes. Nitrocellulose membranes then were blocked in 5% of non-fat milk or 5% of BSA, 10 mmol/L Tris pH 7.5, 100 mM NaCl, 0.1% Tween 20, probed with mouse anti-β-tubulin and mouse anti-β-actin antibodies (Sigma-Aldrich, St. Louis, MO, USA) (primary antibodies dilution 1:10,000), rabbit polyclonal anti-nNOS and anti-iNOS antibodies (primary antibodies dilution 1:200), rabbit polyclonal anti-PARP-1 (primary antibody dilution 1:200), rabbit polyclonal anti-p53 (primary antibody dilution 1:200), mouse monoclonal anti-p21 (primary antibody dilution 1:200), rabbit polyclonal anti-Nrf-2 (primary antibody dilution 1:750) (all purchased by Santa Cruz Biotechnology, CA, USA), rabbit monoclonal anti-Erk 1/2 and anti-phospho-Erk 1/2, rabbit polyclonal anti-p38 and anti-phospho-p38 (all purchased by Cell Signaling Technology, MA, USA) and incubated in the presence of specific enzyme conjugated IgG horseradish peroxidase. Immunoreactive bands were identified using the ECL detection system (LiteAblot Extend Chemiluminescent Substrate, EuroClone S.p.a., Milan, Italy) and analyzed by densitometry. Densitometric values, expressed as Integrated Optical Intensity (IOI), were estimated in the CHEMIDOC XRS system using the QuantiOne 1-D analysis software (BIORAD, Richmond, CA, USA). Values obtained were normalized based on densitometric values of internal β-tubulin or β-actin. Results are expressed as mean ± SD.

### 4.8. MTT Assay

Cell metabolic activity of C6 rat glioma cells was assessed by MTT (3-(4,5-Dimethylthiazol-2-yl)-2,5-Diphenyltetrazolium Bromide) test (Sigma Aldrich, Milan, Italy). Cells were seeded (8 × 10^3^/well) in a 96-well tissue culture-treated plate (Falcon^®^, Corning Incorporated, NY, USA) and let them adhere for 24 h. In a first set of experiments, medium was afterwards removed and C6 rat glioma cells were pre-incubated with TMZ (500 µM) and CM544 (1.5 mM) for 24 h and then exposed to TMZ and/or CM544 for further 24 h. Cell treatment was performed as follow: untreated cells (Control), 24 h in the presence of TMZ or CM544 (TMZ and CM544, respectively), 48 h in the presence of TMZ or CM544 (T1 and T2, respectively) and 24 h of pre-incubation with CM544 followed by additional 24 h of exposition to TMZ and CM544 in parallel (T3). In a second set of experiments, the cell monolayer was incubated in the presence of loading concentrations (0–400 µM) of BSO (L-Buthionine sulfoximine, Sigma-Aldrich, Milano, Italy) for 24, 48 and 72 h, refreshing the medium every 24 h. Finally, in the last set of experiments, cells were incubated with loading concentrations of CM544 (0–1.5 mM) in the presence of BSO 100 µM for 24, 48 and 72 h. After the exposure time, cells were incubated with 100 µL/well of MTT (1 mg/mL) 1:10 with fresh growth medium for 4 h at 37 °C and 5% CO_2_. Finally, the MTT solution was removed and replaced with 100 µL/well of DMSO. Cells were incubated for additional 20 min at 37 °C and 5% CO_2_ and gently swirled for 10 min at room temperature. The optical density was measured at 540 nm by means of a spectrophotometer (Multiskan GO, Thermo Scientific, Monza, Italy). The results were expressed as the percentage of untreated cells (100%) and each experiment was performed three times in triplicate (*n* = 9).

### 4.9. Cell Cycle Analysis

Aiming to measure the proliferation rate of C6 rat glioma cells exposed to TMZ and CM544, cell cycle progression was assessed by flow cytometry. Cells were seeded (2.4 × 10^5^/well) in a 6-well tissue culture-treated plate (Falcon^®^, Corning Incorporated, NY, USA) and were allowed to adhere for 24 h. Then, growth medium was removed and cells were treated as described for the MTT assay. After the exposure time, cells were washed twice with PBS without calcium and magnesium, harvested with Trypsin EDTA 1X (all purchased by EuroClone S.p.a., Milan, Italy) and counted by means of Trypan blue exclusion test (Sigma Aldrich, Milan, Italy). Approximately 5 × 10^5^ cells/experimental condition were fixed with cold ethanol 70% *v*/*v* and kept overnight at 4 °C. Cells were then resuspended in the staining solution made by PBS without calcium and magnesium, 1 mg/mL Propidium Iodide (final concentration 10 μg/mL) and 10 mg/mL RNAse (final concentration 100 µg/mL) and kept overnight at 4 °C. Cell cycle profiles (1 × 10^4^ events/sample) were finally analyzed with a CytoFLEX Flow Cytometer (Beckman Coulter, FL, USA).

### 4.10. Quantification and Statistical Analysis

Statistical analyses were performed using the GraphPad Prism Software 5.0 (La Jolla, CA, USA) and data are expressed as mean values ± standard deviation (SD). After having verified normal distribution of data (one-way ANOVA test), the differences between the median values were performed using a pairwise comparison through the Student’s *t*-test where the minimal *p* value was set at 0.05.

## 5. Conclusions

Glioma is one of the most aggressive tumors and progress has been made in understanding the molecular mechanisms underlying this tumor. However, current treatments remain ineffective. iNOS has been identified as a key regulator of glial transformation molecular downstream and a key oncogenic pathway in gliomas. In our previous work, we demonstrated an intimate relationship between the selective inhibition of the iNOS and the decrease of C6 rat glioma cell proliferation. Results of the present work suggest that iNOS inhibition by CM544 reduces glioma cells proliferation by enhancing PARP-1 cleavage and compromising the adaptive responses of glioma cells, which are involved in chemoresistance. In fact, the co-administration of TMZ and CM544 potentiated TMZ effects on cell viability. It will be important to deepen the role of CM544 in appropriate chemoresistance cell lines, evaluating possible relationships of iNOS inhibition with collateral molecular targets, such as MGMT. Moreover, the co-administration of this new compound with molecules effective on Nrf-2 associated pathways and the metabolism of glutathione (as seen for the BSO co-administration) could be a promising strategy to improve glioma cell response.
